# Large cell morphology, CMYC+ tumour cells, and PD-1+ tumour cell/intense PD-L1+ cell reactions are important prognostic factors in nodal peripheral T-cell lymphomas with T follicular helper markers

**DOI:** 10.1186/s13000-021-01163-7

**Published:** 2021-11-06

**Authors:** Yasuhito Mihashi, Shoichi Kimura, Hiromi Iwasaki, Yumi Oshiro, Yasushi Takamatsu, Shigeto Kawauchi, Shohei Shimajiri, Kenji Ishizuka, Morishige Takeshita

**Affiliations:** 1 Departments of Pathology, Faculty of Medicine, Fukuoka University, 7-45-1 Nanakuma, Jonan-ku, Fukuoka, 814-0180 Japan; 2 Departments of Otolaryngology, Faculty of Medicine, Fukuoka University, 7-45-1 Nanakuma, Jonan-ku, Fukuoka, 814-0180 Japan; 3 Departments of Haematology, Clinical Research Centre, National Hospital Organisation Kyushu Medical Centre, 1-8-1 Jigyohama, Chuo-ku, Fukuoka, 810-8563 Japan; 4 Department of Pathology, Matsuyama Red Cross Hospital, 1 Bunkyo-cho, Matsuyama, 791-0000 Japan; 5 Departments of Internal Medicine, Division of Medical Oncology, Haematology and Infectious Disease, Faculty of Medicine, Fukuoka University, 7-45-1 Nanakuma, Jonan-ku, Fukuoka, 814-0180 Japan; 6 Departments of Pathology, Clinical Research Centre, National Hospital Organisation Kyushu Medical Centre, 1-8-1 Jigyohama, Chuo-ku, Fukuoka, 810-8563 Japan; 7 Department of Pathology, University of Occupational and Environmental Health, Iseigaoko Yahata Nishi-ku, Kitakyushu, Japan; 8 Department of Internal Medicine, Faculty of Medicine, Kagoshima University, Sakuragaoka 8-35-1, Kagoshima, 890-8544 Japan

**Keywords:** AITL, CMYC, PD-1, PD-L1, Peripheral T-cell lymphoma, T follicular helper cell

## Abstract

**Background:**

The clinicopathological characteristics and prognostic factors in nodal peripheral T-cell lymphomas (PTCLs) with two or more T follicular helper markers (TFH+) are not adequately investigated.

**Methods:**

Immunohistologically, we selected 22 patients with TFH+ lymphoma (PTCL-TFH) in 47 of PTCL-not otherwise specified (NOS), and subclassified into large and small cell groups. We compared the two groups with 39 angioimmunoblastic T-cell lymphoma (AITL) and seven follicular T-cell lymphoma (F-TCL) patients. Prognostic factors were analysed by overall survival in patients with three types of TFH+ PTCLs.

**Results:**

Thirteen large cell and nine small cell PTCL-TFH patients had more than two TFH markers including programmed cell death-1 (PD-1). Large cell PTCL-TFH showed frequent CMYC expression in 10 patients (77%), and four of 11 large cell group (36%) had somatic *RHOA* G17V gene mutation by Sanger sequencing. Large cell PTCL-TFH patients showed significantly worse prognosis than those of the small cell group, AITL, and F-TCL (*p* < 0.05). In TFH+ PTCLs, CMYC+ tumour cells, and combined PD-1 ligand 1 (PD-L1) + tumour cells and intense reaction of PD-L1+ non-neoplastic cells (high PD-L1+ cell group) were significantly poor prognostic factors (*p* < 0.05). Combinations of CMYC+ or PD-1+ tumour cells and high PD-L1+ cell group indicated significantly poor prognosis (*p* < 0.01).

**Conclusion:**

Large cell PTCL-TFH indicated poor prognosis in TFH+ PTCLs. These data suggested that CMYC+ tumour cells and intense PD-L1+ cell reaction influenced tumour cell progression in TFH+ PTCLs, and PD-1+ tumour cell/intense PD-L1+ cell reactions may play a role in immune evasion.

## Background

T follicular helper (TFH) cells are mainly located in germinal centres and frequently express CD4, programmed cell death-1 (PD-1), CD10, chemokine (C-X-C motif) ligand (CXCL) 13, BCL6 and inducible T-cell co-stimulator [1]. In peripheral T-cell lymphomas (PTCLs), angioimmunoblastic T-cell lymphoma (AITL) and follicular T-cell lymphoma (F-TCL) are derived from TFH cells and defined by expression of two or more TFH markers (TFH+) [2, 3]. Furthermore, less than half of patients with PTCL-not otherwise specified (NOS) (41%) exhibit more than two TFH markers (PTCL-TFH) [4], and the PTCL-TFH patients show similar incidences of gene mutations of *Ras homolog family member A (RHOA)* G17V and *tet methylcytosine dioxygenase (TET)2* to those of AITL and F-TCL [5]. No prognostic differences have been reported among PTCL-TFH and TFH− PTCL-NOS patients and AITL. In PTCL-NOS, more than 70% large tumour cells and international prognostic index were significant poor prognostic factors (*p* = 0.008, *p* < 0.001, respectively) [6].

Transcription factor CMYC plays a role in tumour cell proliferation and progression in high grade B-cell lymphoma, T-acute lymphoblastic leukaemia (T-ALL) and adult T-cell leukaemia/lymphoma (ATLL) [7–11]. More than 30% CMYC expression in lymphoma cells was a significant prognostic factor in AITL patients (*p* = 0.008), but not in PTCL-NOS [12]. CMYC controls the function of PD-1 ligand 1 (PD-L1), which has immunosuppressive effects and promotes tumour cell growth in mouse and human T-ALL, and in solid tumours [13]. CMYC expression in non-small cell lung cancer significantly correlated with PD-L1, and patients with CMYC+ and PD-L1+ tumour cells had a worse prognosis than other subgroups (*p* < 0.05) [14].

Interactions between PD-1 and PD-L1 play a role in immune suppression during inflammatory processes, and PD-L1 expression induces an immune evasion mechanism exploited by various malignancies [1, 15]. In T/natural killer (NK) cell neoplasia, PD-L1+ tumour cells were frequently found in anaplastic lymphoma kinase (ALK) + and ALK− systemic anaplastic large cell lymphoma (sALCL), occasionally in PTCL-NOS, and rarely in AITL [16–19]. Patients demonstrating combined PD-L1+ tumour cells and intense reaction of PD-L1+ non-neoplastic cells (high PD-L1+ group) showed significantly poorer prognosis compared with the low PD-L1+ group in the above four types of PTCLs [18]. The combination of PD-1+ tumour cells and high PD-L1+ group was related to shorter survival in AITL patients (*p =* 0.051), but not PTCL-NOS [20].

In the current study, we initially selected PTCL-TFH from PTCL-NOS by immunohistology, and subclassified patients into large and small cell groups. We then compared clinicopathological findings of the two groups of PTCL-TFH with those of AITL and F-TCL. The large cell PTCL-TFH patients sometimes had the *RHOA* G17V mutation, which indicated a group with poor prognosis in TFH+ PTCLs [21]. Furthermore, CMYC+ tumour cells and the combination of PD-1+ tumour cells and high PD-L1+ group indicated significantly poor prognostic factors in patients with three types of TFH+ PTCLs by uni- and multivariate analyses. It was highly suggested that histology, CMYC+ or PD-1+ tumour cell/intense PD-L1+ cell reactions were significantly influential on tumour progression and patient prognosis in TFH+ PTCLs.

## Methods

### Patient selection, histological classification and clinical findings

Registered patients were retrieved retrospectively from the Department of Pathology, Fukuoka University, from 1990 to 2019. Histological classification was performed according to the WHO classification in 2017 [2, 22]. Four TFH markers (PD-1, BCL6, CXCL13 and CD10) were examined by immunohistochemistry. There was no difference in overall survival (OS) between patients with two (*n* = 26) and more than three (*n* = 38) TFH markers (*p* = 0.188), and the five-year survivals were 46 and 51%, respectively. Therefore, more than two TFH markers was decided as TFH phenotype. Diffuse infiltrate of atypical CD4+ lymphocytes with more than two TFH markers, neoplastic clear cell nests, prominent proliferation of high endothelial venules and CD21+ dendritic cell nests were main criteria of AITL. Scattered and patchy infiltrates of plasma cells, histiocytes and eosinophils were reference findings of AITL. PTCL-TFH was defined by lacking the typical histological features of AITL and having more than two TFH markers. Follicular TCL (F-TCL) was definite by nodular proliferation of atypical TFH+ lymphocytes and lacing AITL features. 22 patients with nodal PTCL-TFH, 25 nodal TFH− PTCL-NOS, 39 AITL and 7 F-TCL patients were examined in this study. Criteria of small, medium and large tumour cell sizes were in accordance with those of mantle cells, centrocytes and centroblasts in lymphoid follicles. Among PTCLs, the large cell group was characterised by diffusely non-cohesive proliferation of ≥50% large lymphoma cells with distinct nucleoli. The small cell group consisted of predominantly medium-sized (*n* = 7) and small cell (*n* = 20) lymphomas. The small cell group included 10 cases of Lennert lymphoma. Corresponding medical records were reviewed to obtain clinical information, including Ann Arbor stage, treatments and overall survival.

### Histology, immunohistology, and detection of EBV-encoded RNA

Excised tissue specimens were fixed in 10% formalin to generate formalin-fixed and paraffin embedded (FFPE) wax samples and stained with haematoxylin and eosin. Immunohistology was performed on the tumour tissues using the Leica Bond III automated stainer (Leica Biosystems, Buffalo Grove, IL, USA). Antibodies against the following proteins were used: CD3 (PS1, Leica, Newcastle, UK), CD4 (4B12, Leica), CD8 (C81/44B, Leica), CD10 (56C6, Leica), CD25 (interleukin 2 receptor [IL2R], 4C9, Leica), CD30 (BerH2, DakoCytomation, Glostrup, Denmark), PD-1 (NAT105, Abcam, Cambridge, MA), BCL6 (LN22, Leica), CXCL13 (BLC, R&D, Minneapolis, MN), CMYC (Y69, Abcam), MIB1 (MIB1, Dako), PD-L1 (E1L3N, Cell Signaling, Danvers, MA), CD20 (L26, Nichirei, Tokyo), and CD21 (1F8, Dako). Tumour cell counts were semi-quantitatively calculated by two pathologists and percentages of antibody-positive cells were determined (0, 5, and 10%–100% in 10% increments) in over five high power fields [11]. For the four TFH markers, samples with ≥20% labelling of the tumour cells were considered positive [4]. Expression of CMYC, MIB1 and PD-L1 in ≥50% atypical lymphoid cells was estimated as positive (n+) [16], and amount of PD-L1+ histiocytes and dendritic cells in the entire cell populations was scored as follows: R0 (no staining), R1+ (a few cells to < 5%), R2+ (≥ 5% – < 20%) and R3+ (≥ 20%). For the other antibodies, samples with ≥30% labelling of the tumour cells were considered positive. The presence of EBV infection was determined by in situ hybridisation of EBV-encoded RNA (EBERs) + nuclear signals (BOND EBER probe, Leica).

### Quantitative real time polymerase chain reaction

Total RNAs were extracted from FFPE tumour specimens of 42 patients using the NucleoSpin total RNA FFPEXS (Macherey-Nagel, Duren, Germany), according to the manufacturer’s instructions, on a real-time PCR machine (Mini OpticonTM, BioRad, Hercules, CA, USA). All samples were tested for expression of *CMYC* (assay ID: Hs00905030_m1, amplicon size 87 bp) [11]. In addition, samples were analysed for expression of *GUSB* (Hs99999908_m1), *TBP* (Hs00427620_m1), and *ABL1* (Hs00245443_m1), which were used for normalisation in the final analysis.

### Detection of *RHOA* G17V mutation by Sanger sequencing

DNA samples from FFPE tumour tissue were extracted using a GenElute™ Mammalian DNA Miniprep Kit (Sigma-Aldrich, St. Louis, MO, USA). Detection of *RHOA* G17V mutation and wild type were assessed by allele-specific PCR. For *RHOA* amplification, PCR was performed with AmpliTaq gold (Thermo Fisher Scientific, Waltham, MA, USA) using 40 ng genomic DNA, 0.3 μM primers, and 2 μL AmpliTaq gold master mix. A PCR-amplified product of 244 bp, including the codon for the 17th amino-acid, was obtained in 53 patients, and direct sequencing of these products was performed. The coding DNA position 50G > T mutation of the *RHOA* gene predicted change of the wild-type G (Gly) to the mutant type V (Val) [21].

### Statistical analysis

All pairwise comparisons of categorised variables between the histological groups and types of PTCLs were performed using the *χ*^*2*^ or Fisher’s exact test. Of the 93 recruited patients, 87 PTCL patients were examined for clinical outcome. Outcome was determined by calculating cumulative survival from time of diagnosis to date of the last follow up or death. Overall survival (OS) curves were generated using the Kaplan–Meier method with log-rank tests, and analysed by the proportional Hazard model. A *p* value < 0.05 was considered statistically significant. Analyses were performed using software JMP 10 (SAS Institute, Cary, NC, USA).

## Results

### Clinical features

The clinical features and immunohistological findings of 22 patients with PTCL-TFH and 25 with PTCL-NOS, 39 AITL and 7 F-TCL are shown in Table 1. Thirteen of 22 PTCL-TFH patients (59%) were composed of large cell lymphoma and the remaining nine (41%) were small cell. Six of 11 large cell PTCL-TFH patients (55%) showed ≥5000 U/ml sIL2R, which was significantly higher than that observed in small cell PTCL-NOS (13%) and F-TCL (0%) (*p* = 0.008, *p =* 0.039, respectively). Patients in the four groups of TFH+ PTCLs frequently showed advanced clinical stages III and IV.

**Table 1:**
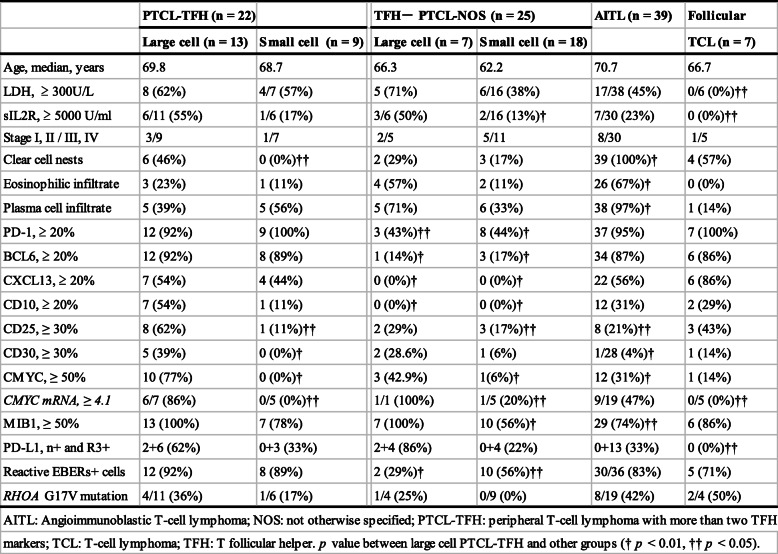
Clinical, histological, immunohistological and genetic findings of 93 patients with nodal peripheral T-cell lymphoma, angioimmunoblastic T-cell lymphoma and follicular T-cell lymphoma

### Histological, immunohistological, and genetic findings, and EBV infection

**Fig. 1:**
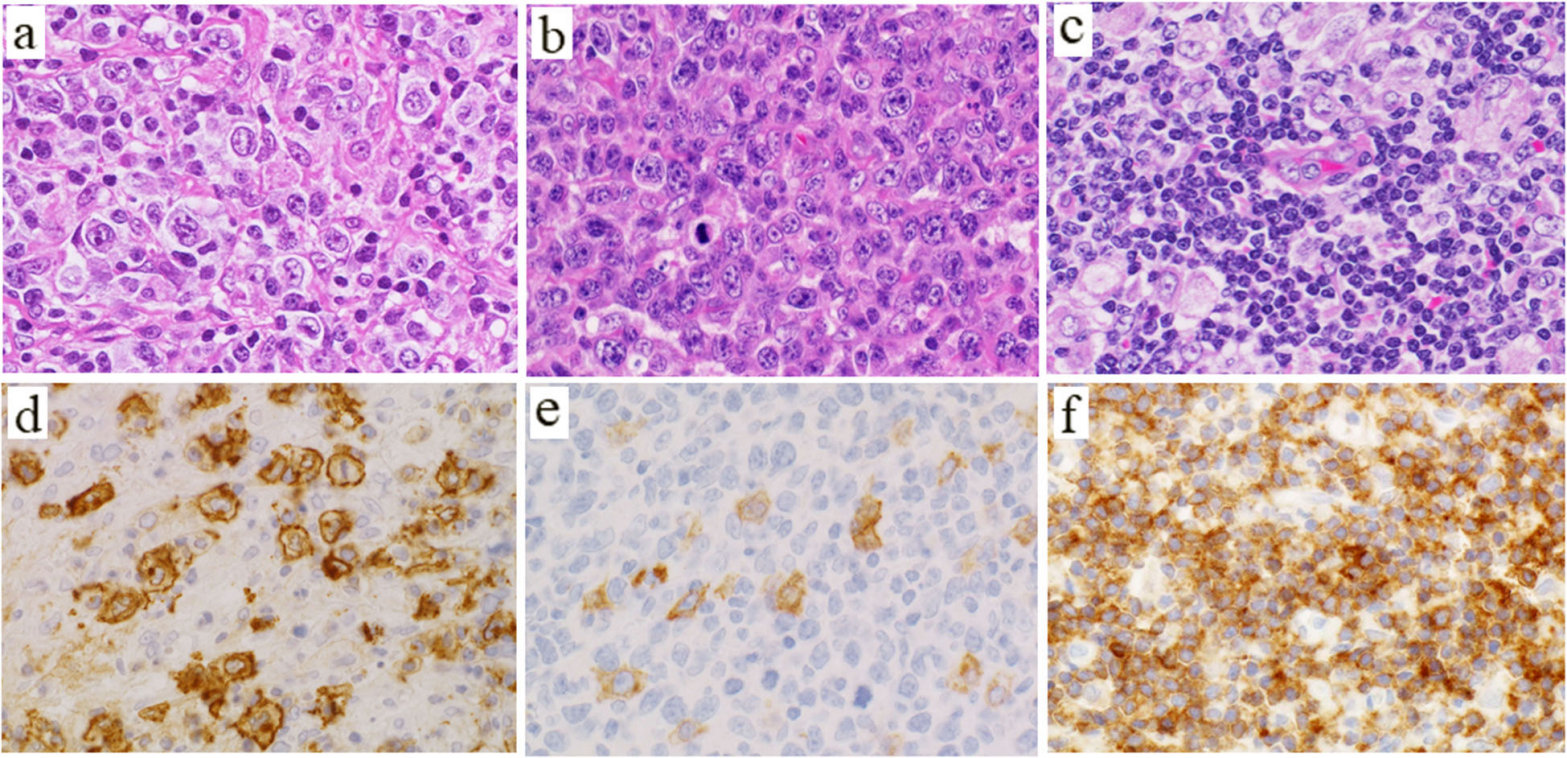
Histological findings and immunohistology in patients with large and small cell PTCL-TFH. Many atypical large lymphocytes are distributed throughout (**a, b**), and small lymphocytes and histiocytes are intermingled in the background (**a**). **c** Diffuse infiltrate of small lymphocytes and nests of epithelioid histiocytes, indicating Lennert lymphoma. **d** Scattered large atypical lymphocytes are positive for CD30, and (**e**) only some CD30+ cells can be seen. **f** Diffuse infiltration of PD-1+ small lymphocytes in Lennert lymphoma. Magnification, × 400

**Fig. 2:**
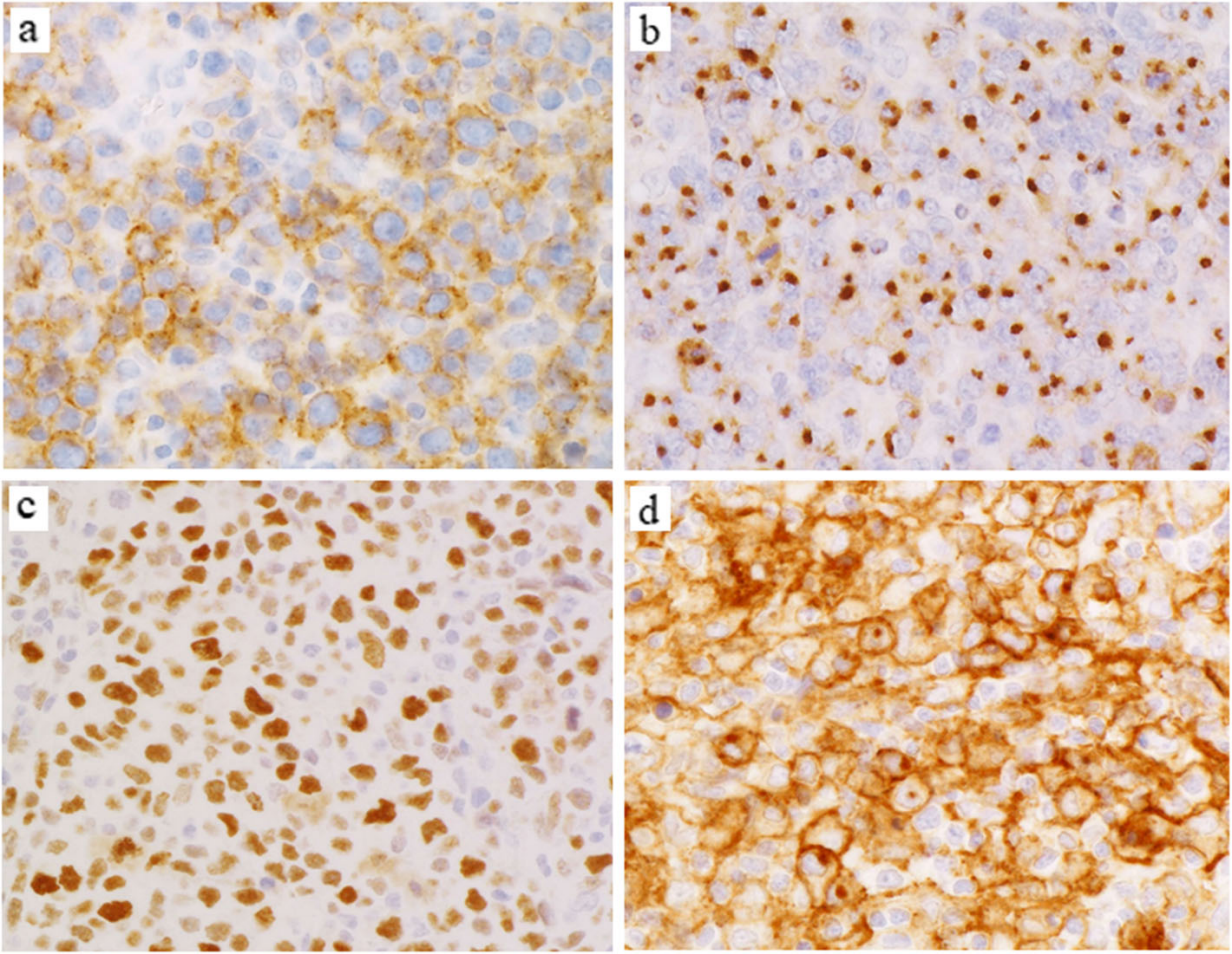
Immunohistological findings of patients with large cell PTCL-TFH. Atypical large lymphoid cells are diffusely positive for PD-1 (**a**), CXCL13 (**b**), CMYC (**c**) and PD-L1 (**d**). PD-L1+ tumour cells, dendritic and histiocytic cells are admixed. Magnification, × 400

Large cell PTCL-TFH patients had significantly lower populations of clear neoplastic cells and fewer reactions of eosinophils and plasma cells than those of AITL (Fig. 1a, b; both *p* < 0.01). Large cell PTCL-TFH expressed more than two TFH markers; among them PD-1 was positive in 12 patients (92%; Fig. 2a), BCL6 in 12 (92%), CXCL13 in seven (54%; Fig. 2b) and CD10 in seven (54%). Small cell PTCL-TFH showed frequent expressions of PD-1 (100%) and BCL6 (89%), but rarely of CD10 (11%). Four of 10 Lennert lymphoma patients showed more than two TFH markers (Fig. 1c, f). Large cell PTCL-TFH showed frequent expression of CD25 in eight patients (62%) and CD30 in five (39%; Fig. 1d, e), which were significantly higher than one (11%) and 0 (0%), respectively, in small cell PTCL-TFH, and eight (21%) and one (4%), respectively, in AITL (*p* < 0.05, *p* < 0.01, respectively). Scattered and patchy infiltrates of CD30+ lymphoma cells were detected in the five large cell PTCL-TFH patients. Large cell PTCL-TFH was frequently positive for CMYC in 10 patients (77%, Fig. 2c) and MIB1 in 13 (100%), which were significantly higher than 0 (0%) and seven (78%), respectively, in small cell PTCL-TFH, and 12 (31%) and 29 (74%), respectively, in AITL (*p <* 0.05, *p <* 0.01, respectively). Mean *CMYC* mRNA was 4.1 in tumour specimens of 42 patients, and ≥ 4.1 *CMYC* mRNA was detected in six of seven large cell PTCL-TFH patients (86%), which was significantly higher than in small cell PTCL-TFH (0/5, 0%) and F-TCL (0/5, 0%) (both *p* < 0.05). The combination of PD-L1+ tumour cells and intense reaction (R3+) of PD-L1+ non-neoplastic cells (high PD-L1+ group) was found in eight large cell PTCL-TFH patients (62%), which was significantly higher than in F-TCL (0%) (*p* = 0.036; Fig. 2d). Scattered EBERs+ lymphocytes in the background were found in large cell (92%) and small cell (89%) PTCL-TFH patients, compared with large cell (29%) and small cell (56%) PTCL-NOS groups (*p* < 0.01, *p* < 0.05, respectively).

### *RHOA* p.G17V mutation by Sanger sequencing

**Fig. 3:**
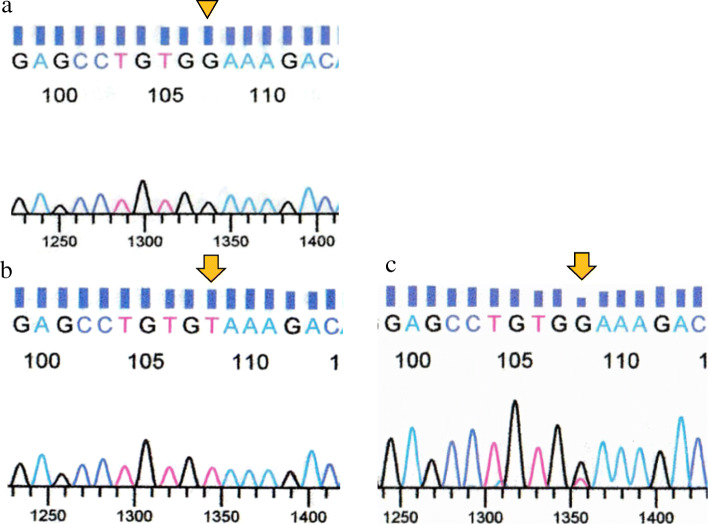
Chromatograms of Sanger sequencing for *RHOA* G17V mutation in patients with AITL (**a**, **b**) and large cell PTCL-TFH (**c**). Arrow head indicates wild type (**a**), and arrows indicate 50G > T mutation (**b, c**)

By Sanger sequencing, four of 11 large cell PTCL-TFH patients (36%), one of six small cell PTCL-TFH (17%), eight of 19 of AITL (42%), two of four F-TCL (50%) and one of 13 PTCL-NOS (8%) patients showed the *RHOA* p.G17V mutation (Fig. 3), but no significant differences were found among the groups.

### Treatments, prognosis and prognostic factors in TFH+ PTCLs

**Fig. 4:**
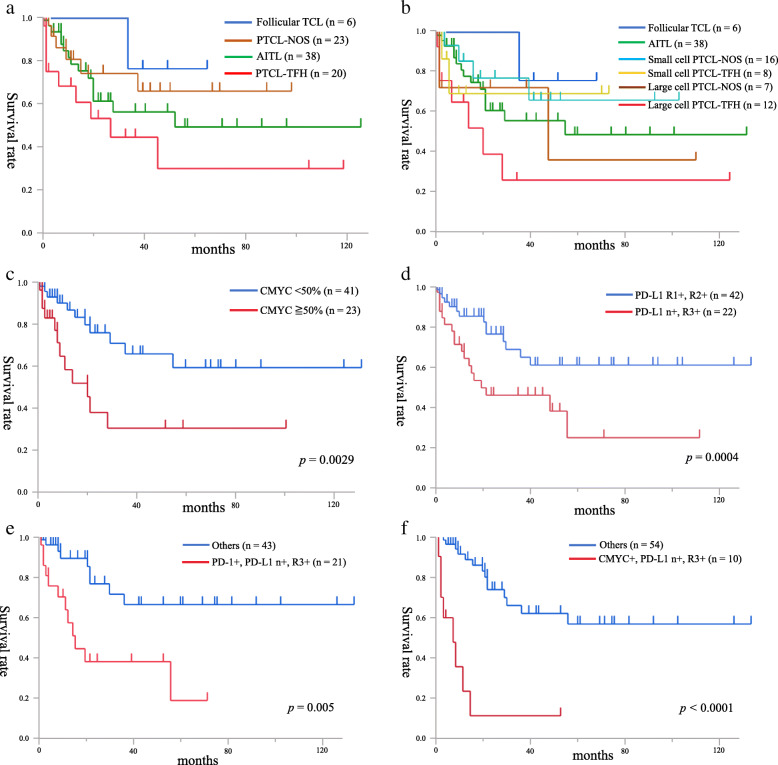
**a** No significant differences in overall survival (OS) were detected among 20 PTCL-TFH patients, 23 PTCL-NOS, and 38 AITL. **b** 12 large cell PTCL-TFH patients show significantly worse OS than small cell PTCL-TFH (*n* = 8; *p =* 0.045), AITL (*n* = 38; *p =* 0.047) and F-TCL (*n* = 6; *p =* 0.012). **c** CMYC+ tumour cells (*n* = 23) are a poor prognostic factor in 64 patients with TFH+ PTCLs (*p =* 0.029). **d** 22 patients with both PD-L1+ tumour cells and R3+ PD-L1+ cells show significantly worse OS than 42 with R1+ and R2+ PD-L1+ cells (*p =* 0.0004). **e** 21 patients with both PD-1+ tumour cells and high PD-L1+ group (*n* + and R3+) show significantly worse prognosis than 43 of the other group (*p =* 0.005). **f** 10 patients with both CMYC+ tumour cells and high PD-L1+ group show significantly poorer OS compared with 54 patients in the other groups of TFH+ PTCLs (*p <* 0.0001)

Of the total cohort, we examined treatments and outcome in 87 patients. Chemotherapies including cyclophosphamide, doxorubicin, vincristine, and prednisone (CHOP) or pirarubicin, cyclophosphamide, vincristine, and prednisolone (THP-COP) were mainly administrated in the all examined patients. Nine of 20 PTCL-TFH patients (45%), seven of 23 PTCL-NOS (30%), 15 of 38 AITL (40%) and one of six F-TCL (17%) died of disease. The examined PTCL-TFH, PTCL-NOS, AITL and F-TCL patients showed no prognostic differences between groups (Fig. 4a). Twelve patients with large cell PTCL-TFH showed significantly poorer OS than eight small cell PTCL-TFH, 16 small cell PTCL-NOS, 38 AITL and six F-TCL patients (*p* = 0.045, *p* = 0.014, *p* = 0.047 and *p* = 0.012, respectively; Fig. 4b). Table 2 shows the univariate analysis of risk factors for OS in the examined 64 patients with TFH+ PTCLs. In TFH+ PTCLs, ≥ 300 IU/L lactate dehydrogenase (LDH; *n* = 28) and clinical stages III and IV (*n* = 50) were significant poor prognostic factors (*p* = 0.04, *p* = 0.022, respectively). Presence of CMYC+ tumour cells (*n* = 23) was a significantly poor OS factor (*p* = 0.029; Fig. 4c). Presence of the high PD-L1+ group (*n* = 23) showed a significantly poor OS compared with presence of the low (R1+, R2+) PD-L1+ group (*n* = 41) (*p* = 0.0004; Fig. 4d). Furthermore, 21 patients with the combination of PD-1+ tumour cells and high PD-L1+ group showed significantly poorer OS than the other groups (*n* = 43) (*p* = 0.005; Fig. 4e). The combination of CMYC+ tumour cells and high PD-L1+ group (*n* = 10) also indicated prominently poor OS compared with the other groups (*n* = 54) (*p* < 0.0001; Fig. 4f). The *RHOA* p.G17V mutation was not a prognostic factor in examined 40 patients with TFH+ PTCLs. Multivariate analysis indicated that presence of CMYC+ tumour cells (*p* = 0.039) and high PD-L1+ group (*p* = 0.001) were significantly associated with OS in 64 patients with TFH+ PTCLs in Table 2.

**Table 2:**
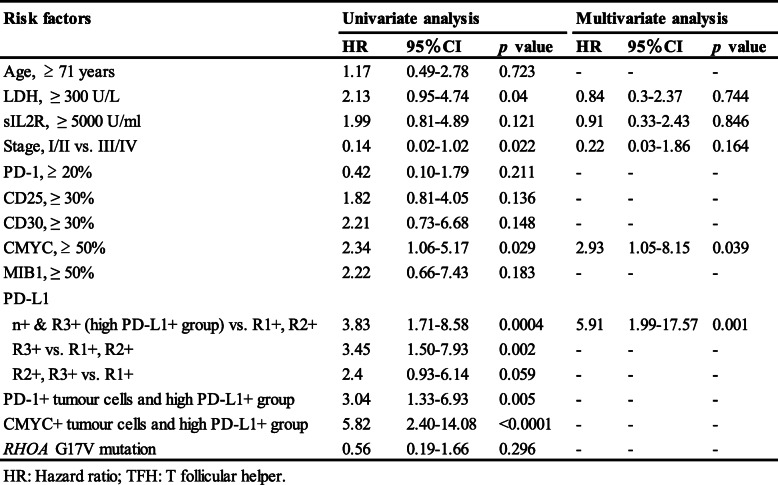
Univariate and multivariate analyses of risk factors for overall survival in examined 64 patients with TFH+peripheral T-cell lymphomas

## Discussion

The current study demonstrated that 22 of 47 PTCL-NOS patients (47%) showed more than two TFH markers, and no prognostic differences were found among patients with AITL, PTCL-TFH and PTCL-NOS. Other researchers have reported that lymphoma cells in 18 of 41 PTCL-NOS patients (44%) showed more than two TFH markers [23]. Although BCL6 expression and *RHOA* G17V mutation were significantly higher in AITL patients than in PTCL-NOS (both *p* < 0.01), neither definite clinical nor prognostic differences were found among above three groups of PTCLs. In our examined TFH+ PTCLs, large cell PTCL-TFH patients had a similar incidence of *RHOA* G17V mutation (36%) to those with AITL (42%), but showed higher expressions of CD25 (IL2R), CD30, CMYC, and *CMYC* mRNA, than small cell PTCL-TFH patients and AITL (*p <* 0.05 or *p <* 0.01, respectively). Furthermore, large cell PTCL-TFH patients showed significantly poorer OS compared with small cell PTCL-TFH, AITL and F-TCL (all *p* < 0.05). Other researchers have reported that CD30+ giant cells or large tumour cells were detected in 64 of 217 PTCL-NOS patients (32%) and six of 25 PTCL-TFH cases (24%) [3, 6]. CD30 as well as CD25 (IL2R) may be one of the activation molecules in large cell PTCL-TFH. Patients with F-TCL were frequently found to be in advanced clinical stages III and IV, but showed relatively indolent prognosis compared with AITL [24, 25]. These findings, together with our results demonstrated that the large cell morphology of PTCL-TFH indicated distinct pathological and immunohistological features and poor prognosis in groups of patients with TFH+ PTCLs.

CMYC protein and mRNA expressions in aggressive type ATLL patients were significantly higher than those of smouldering and chronic types (*p* < 0.01), and CMYC may accelerate the conversion from indolent to aggressive type ATLL [11]. In the current study, CMYC+ tumour cells were frequently detected in 10 of 13 large cell PTCL-TFH patients (77%), and was a significantly poor prognostic factor in patients with TFH+ PTCLs by the uni- and multivariate analyses (*p* = 0.029, *p* = 0.039, respectively). Other researchers have reported that expressions of CMYC and Th2-cell transcription factor GATA3 were frequently found in 128 nodal PTCL patients with AITL, PTCL-NOS and sALCL, and CMYC+ tumour cells indicated significantly poor prognosis in the above three types of PTCL and in only AITL (all *p* < 0.01) [12]. GATA3+ PTCL-NOS patients showed frequent copy number gains/amplifications of *CMYC* and *STAT3*, and loss of *CDKN2A*, having inferior OS compared with the GATA3− group [7]. CMYC and GATA3 may be important transcription factors that significantly affect the prognosis of patients with TFH+ PTCLs.

Sun et al. reported that the presence of the high PD-L1+ group (46%) including PD-L1+ tumour cells, resulted in significantly poorer prognosis compared with presence of the low PD-L1+ group (54%) in 144 patients with AITL, PTCL-NOS, ALK+ and ALK− sALCL (*p* < 0.05) [18]. However, PD-L1+ tumour cells were frequently found in 34 of 45 ALK+ (76%) and 21 of 50 ALK− (42%) sALCL patients [19]. In addition, sALCL also showed less frequent expression of TFH markers [26, 27]. The results supported sALCL as a distinct disease from AITL and PTCL-NOS. In the current study, presence of the high PD-L1+ group was a significant poor prognostic factor in 64 patients with TFH+ PTCLs by the uni- and multivariate analyses (*p* = 0.0004, *p* = 0.001, respectively). Furthermore, the combination of PD-1+ tumour cells and high PD-L1+ group also indicated significantly poor prognosis (*p* = 0.005). A recent report showed that the combination of PD-1+ tumour cells and the high PD-L1+ group significantly correlated with elevated serum LDH in AITL and PTCL-NOS patients (*p* = 0.03), and was related to shorter OS in patients with AITL (*p =* 0.051), being significant in clinical stage IV of AITL (*p =* 0.007) [20]. It was highly suggested that the combination of PD-1+ tumour cells and PD-L1+ cells induced intrinsic immune escape and poor prognosis in patients with TFH+ PTCLs.

JQ1, a bromodomain and extra-terminal protein (BET) inhibitor, blocks acetylation of N-terminal histone tails and suppresses tumour initiating cells. JQ1 treatment resulted in growth arrest and apoptosis in mouse and human T-ALL and solid tumour cells due to CMYC inactivation and immune reactivation by downregulation of PD-L1 [28, 29]. CMYC and PD-L1 double expression in pancreas cancer in 87 patients was significantly associated with poor histological grade and poor OS (*p* < 0.01) [30]. Furthermore, JQ1 combined with anti-PD-L1 treatment suppressed both CMYC and PD-L1 in cancer cell lines and mouse models, and exerted synergistic inhibition of pancreas cancer growth. In the current study, the combination of CMYC+ tumour cells and high PD-L1+ group was a significant poor prognostic factor in TFH+ PTCLs (*p* < 0.0001). Inactivation of CMYC pathways and immune reactivation by downregulation of PD-L1 may be effective therapeutic strategies for tumour cell reduction in patients with TFH+ PTCLs.

In conclusion, differentiating large and small cell PTCL-TFH is necessary. Large cell PTCL-TFH patients showed frequent expression of activation molecules and sometimes *RHOA* G17V mutation, and pursued a progressive clinical course in groups of TFH+ PTCLs. Presence of CMYC+ tumour cells or the high PD-L1+ group, and the combination of these two were significantly poor prognostic factors in patients with TFH+ PTCLs (*p* = 0.029, *p* = 0.0004, or *p* < 0.0001, respectively). Furthermore, the combination of PD-1+ tumour cells and high PD-L1+ group induced significantly poor prognosis (*p* = 0.005). CMYC inactivation and immune checkpoint inhibitors might improve patient prognosis in TFH+ PTCLs. Further study is necessary to confirm the clinicopathological characteristics of PTCL-TFH because of the small number of patients in the current study.

## Data Availability

The datasets used and analysed in the current study are available from the corresponding author on reasonable request.

## Abbreviations

AITL: Angioimmunoblastic T-cell lymphoma
ALK: Anaplastic lymphoma kinase
ALL: Acute lymphoblastic leukaemia
ATLL: Adult T-cell leukaemia/lymphoma
EBV: Epstein-Barr virus
EBER: EBV-encoded RNA
F-TCL: Follicular T-cell lymphoma
NOS: Not otherwise specified
PD-1: Programmed cell death-1
PD-L1: Programmed cell death-1 ligand 1
PTCL: Peripheral T-cell lymphoma
TFH: T follicular helper
